# Development and internal validation of a nomogram for predicting postoperative hypotension after carotid endarterectomy

**DOI:** 10.3389/fsurg.2026.1910322

**Published:** 2026-08-28

**Authors:** Jia Zheng, Sensen Wu, Lianrui Guo

**Affiliations:** Department of Vascular Surgery, Xuanwu Hospital, Capital Medical University, Beijing, China

**Keywords:** carotid endarterectomy, LASSO regression, nomogram, postoperative hypotension, risk prediction

## Abstract

**Objectives:**

Postoperative hypotension is one of the common and serious complications following carotid endarterectomy (CEA). Early identification of high-risk patients is significance for improving perioperative management. This study aims to develop and validate a nomogram model for predicting post-CEA hypotension.

**Methods:**

This single-center retrospective study included 527 patients with carotid artery stenosis who underwent CEA. They were randomly divided into a training cohort (*n* = 369) and an internal validation cohort (*n* = 158) at a ratio of 7:3. The demographic characteristics, clinical data and perioperative data of patients were collected in the hospital medical record system. The least absolute shrinkage and selection operator (LASSO) regression was used for predictor selection, and a postoperative hypotension risk prediction model was developed based on multivariable logistic regression, which was then visualized as a nomogram. The discrimination, calibration, and clinical utility of the model were evaluated using receiver operating characteristic (ROC) curves, calibration curves, and decision curve analysis (DCA).

**Results:**

Postoperative hypotension occurred in 88 of 527 patients (17%). Eight predictors were included in the nomogram model, including plaque length above the carotid bifurcation, diabetes mellitus, hypertension, symptomatic stenosis, contralateral carotid stenosis, homocysteine, operation duration, and surgical treatment. The model achieved good discrimination in the training and internal validation cohorts, with AUCs of 0.864 (95% CI, 0.814–0.914) and 0.868 (95% CI, 0.792–0.944), respectively, and showed good calibration and clinical utility.

**Conclusion:**

This study developed and validated a nomogram model for predicting post-CEA hypotension, which can provide a reference for patient risk assessment and individualized perioperative management.

## Introduction

Carotid artery stenosis is a common atherosclerotic vascular disease. As one of the major causes of ischemic stroke, CAS accounts for approximately 20%–25% of all cases (1). A population-based study in the United States estimated that approximately 7% of adults older than 65 years have moderate-to-severe carotid artery stenosis, and asymptomatic CAS is particularly common among older adults (2). Carotid endarterectomy is an effective revascularization procedure for the treatment of carotid artery stenosis. However, perioperative complications, particularly hemodynamic instability, remain important factors affecting postoperative outcomes. Denervation or dysfunction of carotid sinus baroreceptors during surgery is considered one of the major pathophysiological mechanisms underlying postoperative blood pressure changes (3). Surgical manipulation near the carotid sinus may reduce baroreflex sensitivity and cause autonomic imbalance, including excessive sympathetic activation, thereby contributing to postoperative blood pressure instability. Postoperative changes in cerebral hemodynamics may further contribute to blood pressure instability after CEA, and previous studies have shown that patients undergoing CEA may experience marked hemodynamic fluctuations, including both hypertensive and hypotensive events (4). Among these complications, postoperative hypotension deserves particular attention because it occurs in approximately 10% of patients and may lead to adverse events such as cerebral ischemia and organ hypoperfusion (5). Several studies have suggested that postoperative hypotension after CEA may be associated with preoperative clinical status and surgical technique (6, 7). However, effective tools for individualized prediction are still lacking, limiting early identification of high-risk patients and timely clinical intervention. A nomogram is a visual statistical tool that integrates multiple predictors to provide individualized risk estimation and facilitate clinical decision-making. Nomograms have been widely used in the clinical assessment of various diseases and have shown good utility in risk prediction (8). Therefore, this study aimed to develop and internally validate a prediction model for postoperative hypotension in patients undergoing CEA, in order to rapidly identify high-risk patients and provide a basis for timely individualized perioperative management, thereby reducing hypotension-related adverse events and improving patient outcomes.

## Patients and methods

### Patient sample and data collection

This single-center retrospective study was conducted at the Department of Vascular Surgery, Xuanwu Hospital, Capital Medical University. A total of 527 patients with carotid artery stenosis who underwent CEA over a three-year period were included. Clinical data were retrieved from the hospital electronic medical record system. Demographic and clinical variables included age, sex, hypertension, diabetes mellitus, coronary heart disease, smoking history, and alcohol consumption. Laboratory variables included homocysteine, total cholesterol, triglycerides, low-density lipoprotein cholesterol, and high-density lipoprotein cholesterol. All patients underwent preoperative cervical vascular Doppler ultrasonography, from which plaque length, plaque extension above and below the carotid bifurcation, original and residual carotid artery diameter, blood flow velocity, and the presence of ulcerative plaque were recorded. Operative variables, including surgical technique, operative duration, shunting, and intraoperative blood loss, were also collected. Baseline blood pressure was measured after admission. Following surgery, all patients were admitted to the departmental intensive care unit. Blood pressure was continuously monitored for the first 24 h and then measured four times daily until postoperative day 3 or hospital discharge. Postoperative hypotension was defined as either (1) a sustained decrease in systolic blood pressure below 90 mmHg or mean arterial pressure below 65 mmHg for more than 15 min, or (2) the need for continuous intravenous infusion of a vasoactive medication for more than 15 min to maintain blood pressure above these thresholds, consistent with the definition used in previous studies (5). This definition was applied retrospectively based on electronic medical record data and continuous blood pressure monitoring records collected during the first 24 h postoperatively and subsequent four-times-daily measurements until postoperative day 3.

### Inclusion and exclusion criteria

Patients diagnosed with carotid artery stenosis who underwent carotid endarterectomy (CEA) at Xuanwu Hospital, Capital Medical University, during the study period were eligible for inclusion. Patients were excluded if they had incomplete perioperative clinical, laboratory, imaging, or postoperative blood pressure monitoring data; underwent concomitant major surgical procedures; had a history of previous ipsilateral carotid surgery or repeat CEA; or experienced perioperative death or severe complications that precluded postoperative outcome assessment.

### Carotid endarterectomy

All carotid endarterectomy (CEA) procedures were performed under general anesthesia with endotracheal intubation. The operations were conducted by three surgeons from the same team with comparable surgical expertise. The surgical technique was selected by the operating surgeon according to lesion characteristics, vascular anatomy, and intraoperative judgment. The decision to use an intraoperative shunt was based on the reduction in internal carotid artery blood flow after carotid clamping. Shunting was performed when blood flow decreased by more than 50% compared with the pre-clamping baseline.

### Statistical analysis

All statistical analyses were performed using SPSS (version 26.0) and R software (version 4.2.2). Patients were randomly divided into a training cohort and an internal validation cohort at a ratio of 7:3. Baseline characteristics were compared between the two cohorts. Continuous variables were presented as mean ± standard deviation (SD) or median (interquartile range, IQR), as appropriate, and were compared using the independent-samples t test or Mann–Whitney *U* test. Categorical variables were expressed as counts and percentages and were compared using the chi-square test or Fisher's exact test. In the training cohort, LASSO regression analysis was performed for preliminary variable selection. The selected variables were subsequently entered into a multivariable logistic regression model for model development, and the resulting model was visualized as a nomogram. All variables selected by LASSO with non-zero coefficients were entered into the multivariable logistic regression model and retained in the final nomogram, consistent with the principle that prediction models prioritize overall predictive performance over individual variable significance. The predictive performance of the nomogram was assessed in both the training and internal validation cohorts. Discrimination was evaluated using the area under the receiver operating characteristic curve, calibration was assessed using calibration curves, and clinical usefulness was evaluated by decision curve analysis (9). Results with a *p*-value < 0.05 were considered statistically significant.

## Results

### Study population

A total of 527 patients with carotid artery stenosis who underwent carotid endarterectomy were enrolled in this study and randomly assigned to a training cohort (*n* = 369) and an internal validation cohort (*n* = 158) in a 7:3 ratio. Postoperative hypotension occurred in 88 of 527 patients (17%), including 70 of 369 patients (19%) in the training cohort and 18 of 158 patients (11%) in the internal validation cohort. Baseline characteristics of the training and internal validation cohorts are shown in Table 1. Most variables were comparable between the two cohorts, although differences were observed in original carotid artery diameter, peak systolic velocity (PSV), surgical treatment, and operative duration.

**Table 1:** Baseline characteristics in training and internal validation cohorts.

Characteristic	Cohort		*p*-value
	Training cohort *N* = 369	Internal validation cohort *N* = 158	
Sex, *n* (%)			0.633^a^
Female	48 (13.0%)	23 (14.6%)	
Male	321 (87.0%)	135 (85.4%)	
Age, *n* (%)			0.996^a^
<70	285 (77.2%)	122 (77.2%)	
≥70	84 (22.8%)	36 (22.8%)	
Symptom, *n* (%)			0.449^a^
No	214 (58.0%)	86 (54.4%)	
Yes	155 (42.0%)	72 (45.6%)	
Hypertension, *n* (%)			0.600^a^
No	175 (47.4%)	71 (44.9%)	
Yes	194 (52.6%)	87 (55.1%)	
Diabetes mellitus, *n* (%)			0.618^a^
No	244 (66.1%)	108 (68.4%)	
Yes	125 (33.9%)	50 (31.6%)	
Coronary artery disease, *n* (%)			0.650^a^
No	296 (80.2%)	124 (78.5%)	
Yes	73 (19.8%)	34 (21.5%)	
Percutaneous coronary intervention, *n* (%)			0.188^a^
No	344 (93.2%)	142 (89.9%)	
Yes	25 (6.8%)	16 (10.1%)	
Smoking, *n* (%)			0.962^a^
No	193 (52.3%)	83 (52.5%)	
Yes	176 (47.7%)	75 (47.5%)	
Drinking, *n* (%)			0.378^a^
No	255 (69.1%)	103 (65.2%)	
Yes	114 (30.9%)	55 (34.8%)	
Homocysteine umol/L, Mean ± SD	17 ± 10	16 ± 9	0.215^b^
TG mmol/L, Mean ± SD	3.67 ± 1.00	3.56 ± 0.89	0.247^b^
LDL mmol/L, Mean ± SD	2.03 ± 0.80	1.95 ± 0.71	0.234^b^
HDL mmol/L, Mean ± SD	1.13 ± 0.33	1.11 ± 0.34	0.572^b^
Side, *n* (%)			0.121^a^
Left side	169 (45.8%)	84 (53.2%)	
Right side	200 (54.2%)	74 (46.8%)	
Stenosis grade, *n* (%)			0.703^a^
Moderate	85 (23.0%)	34 (21.5%)	
Severe	284 (77.0%)	124 (78.5%)	
Ulcerative plaque, *n* (%)			0.703^a^
No	295 (79.9%)	124 (78.5%)	
Yes	74 (20.1%)	34 (21.5%)	
Plaque length mm, Mean ± SD	30 ± 9	32 ± 11	0.076^b^
Above the fork mm, Mean ± SD	17 ± 7	17 ± 8	0.435^b^
Below the fork mm, Mean ± SD	13 ± 8	15 ± 9	0.107^b^
Residual diameter mm, Mean ± SD	1.36 ± 0.53	1.40 ± 0.56	0.449^b^
Original diameter mm, Mean ± SD	6.97 ± 1.24	7.37 ± 1.22	<0.001^b^
ICA diameter mm, Mean ± SD	3.99 ± 1.14	4.11 ± 1.05	0.237^b^
PSV, Mean cm/s ± SD	386 ± 159	417 ± 162	0.044^b^
External carotid artery stenosis, *n* (%)			0.742^a^
No	261 (70.7%)	114 (72.2%)	
Yes	108 (29.3%)	44 (27.8%)	
Contralateral carotid stenosis, *n* (%)			0.830a^b^
No	277 (75.1%)	120 (75.9%)	
Yes	92 (24.9%)	38 (24.1%)	
Surgical treatment, *n* (%)			0.024^a^
Eversion	163 (44.2%)	51 (32.3%)	
Patch	125 (33.9%)	59 (37.3%)	
Tradition	81 (22.0%)	48 (30.4%)	
Shunting, *n* (%)			0.175^a^
No	293 (79.4%)	117 (74.1%)	
Yes	76 (20.6%)	41 (25.9%)	
Operation duration, Mean ± SD	143 ± 41	152 ± 51	0.043^b^
Lose blood, Mean ± SD	44 ± 46	56 ± 75	0.071^b^

a Pearson's Chi-squared test.

b Welch Two Sample *t*-test.

### Predictor selection and multivariable model construction

**Figure 1:**
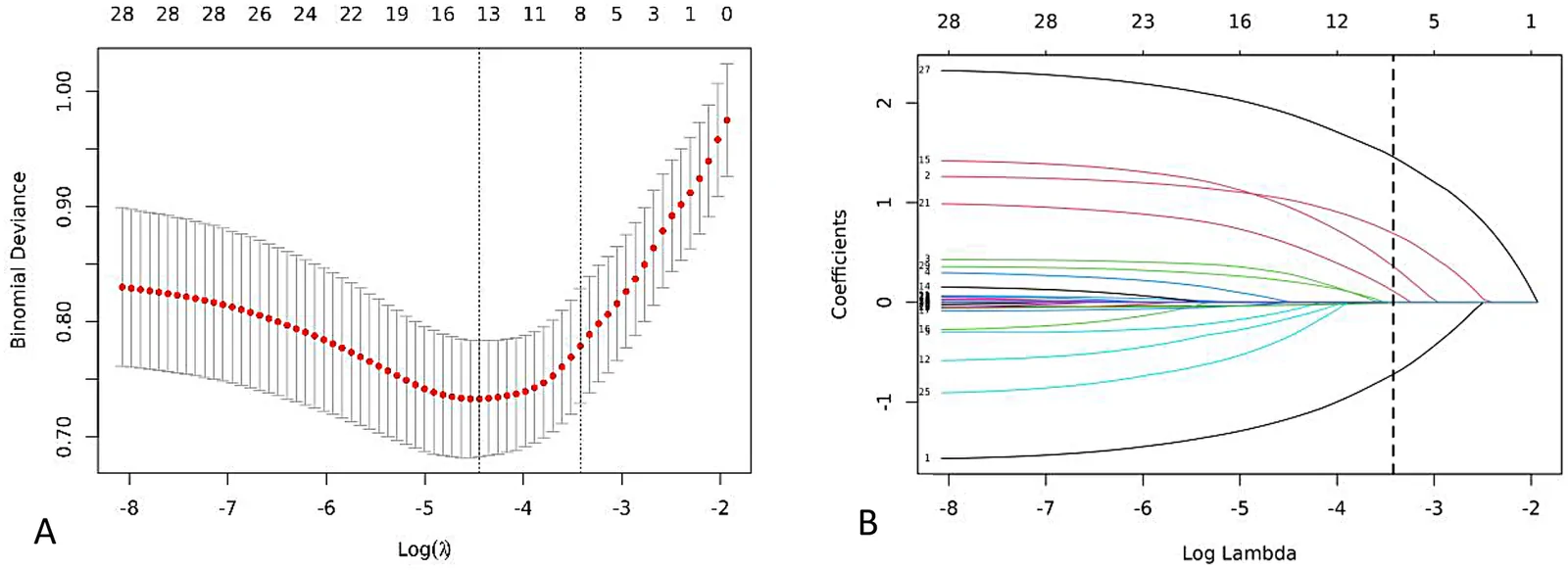
**(A)** Plot for LASSO regression coefficients. **(B)** Cross-validation plot for the penalty term.

Variables in the training cohort were first entered into the LASSO regression model for feature selection to identify predictors of postoperative hypotension. Using cross-validation, the optimal penalty parameter was determined, and eight variables with non-zero coefficients were retained as candidate predictors. The coefficient profiles of the selected variables are shown in Figure 1A, and the cross-validated error plot of the LASSO regression model is presented in Figure 1B. These candidate variables were subsequently entered into the multivariable logistic regression model (Table 2). To further assess the impact of the non-significant predictors (plaque extension above the bifurcation and operation duration), we performed a sensitivity analysis by comparing the full model (eight variables) with a reduced model excluding these two variables. The reduced model achieved an AUC of 0.858 (95% CI, 0.806–0.911) in the training cohort and 0.845 (95% CI, 0.756–0.934) in the internal validation cohort, compared with 0.864 (95% CI, 0.814–0.914) and 0.868 (95% CI, 0.792–0.944) for the full model, respectively. The decrease in AUC was more pronounced in the validation cohort (Δ = 0.023) than in the training cohort (Δ = 0.006), suggesting that these two variables, although not statistically significant individually, contributed to the model's generalizability. All variables selected by LASSO were entered into the final multivariable model and retained in the nomogram for prediction, although some variables did not reach conventional statistical significance.

**Table 2:** Multivariable logistic regression analysis for postoperative hypotension in the training cohort.

Characteristic	*N*	Event N	OR	95% CI	*p*-value
Symptom					
No	214	34	—	—	
Yes	155	36	3.89	1.90, 7.98	<0.001
Hypertension					
No	175	48	—	—	
Yes	194	22	0.21	0.10, 0.43	<0.001
DM					
No	244	31	—	—	
Yes	125	39	3.67	1.86, 7.24	<0.001
Homocysteine	369	70	0.95	0.91, 0.99	0.009
Above the fork	369	70	0.95	0.91, 1.00	0.060
Contralateral carotid stenosis					
No	277	46	—	—	
Yes	92	24	2.35	1.14, 4.81	0.020
Surgical treatment					
Eversion	163	14	—	—	
Patch	125	49	12.22	5.24, 28.48	<0.001
Tradition	81	7	1.50	0.51, 4.47	0.464
Operation duration	369	70	1.01	1.00, 1.01	0.107

CI, confidence interval; OR, odds ratio. Null deviance = 359; Null df = 368; Log-likelihood = −121; AIC = 262; BIC = 301; Deviance = 242; Residual df = 359; No. Obs. = 369.

### Development of the nomogram

**Figure 2:**
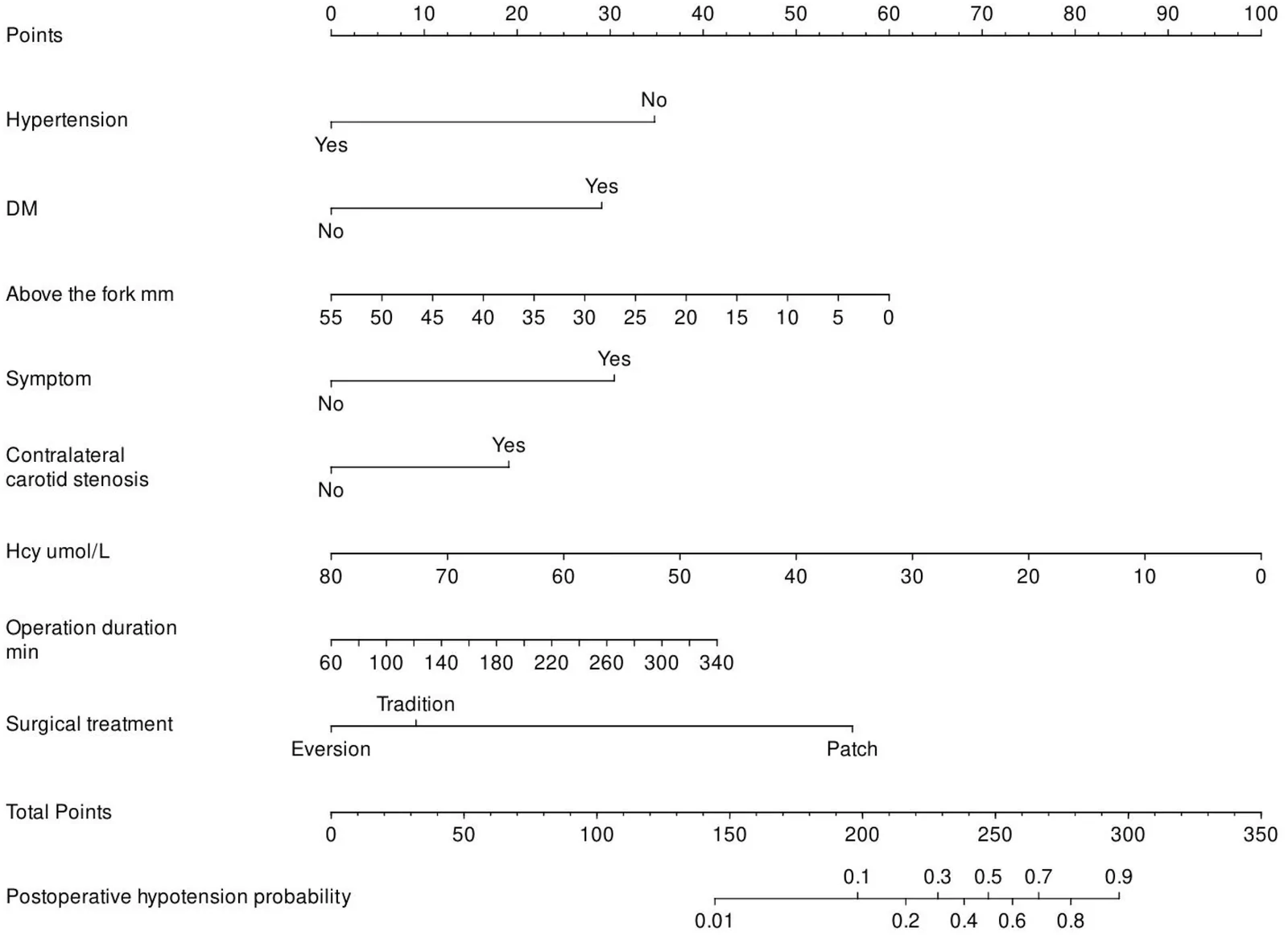
Nomogram developed to estimate the individualized risk of postoperative hypotension after carotid endarterectomy.

Based on the final prediction model, a nomogram was developed to estimate the individualized risk of postoperative hypotension after carotid endarterectomy. The nomogram incorporated eight predictors selected by LASSO regression, including hypertension, diabetes mellitus, plaque extension above the carotid bifurcation, symptomatic status, contralateral carotid stenosis, homocysteine, operation duration, and surgical treatment (Figure 2).

In the nomogram, each predictor was assigned a weighted score according to its relative contribution to the model. By summing the points for all variables, a total score was obtained and then converted into the corresponding predicted probability of postoperative hypotension. Higher total points indicated a greater risk of postoperative hypotension. Based on the Youden index, the optimal cutoff value for identifying high-risk patients was a total score of ≥230 points, corresponding to a predicted probability of 26%, with a sensitivity of 73% and a specificity of 63%. The nomogram provided an intuitive and individualized tool for perioperative risk assessment in patients undergoing carotid endarterectomy.

### Validation and clinical utility of the nomogram

**Figure 3:**
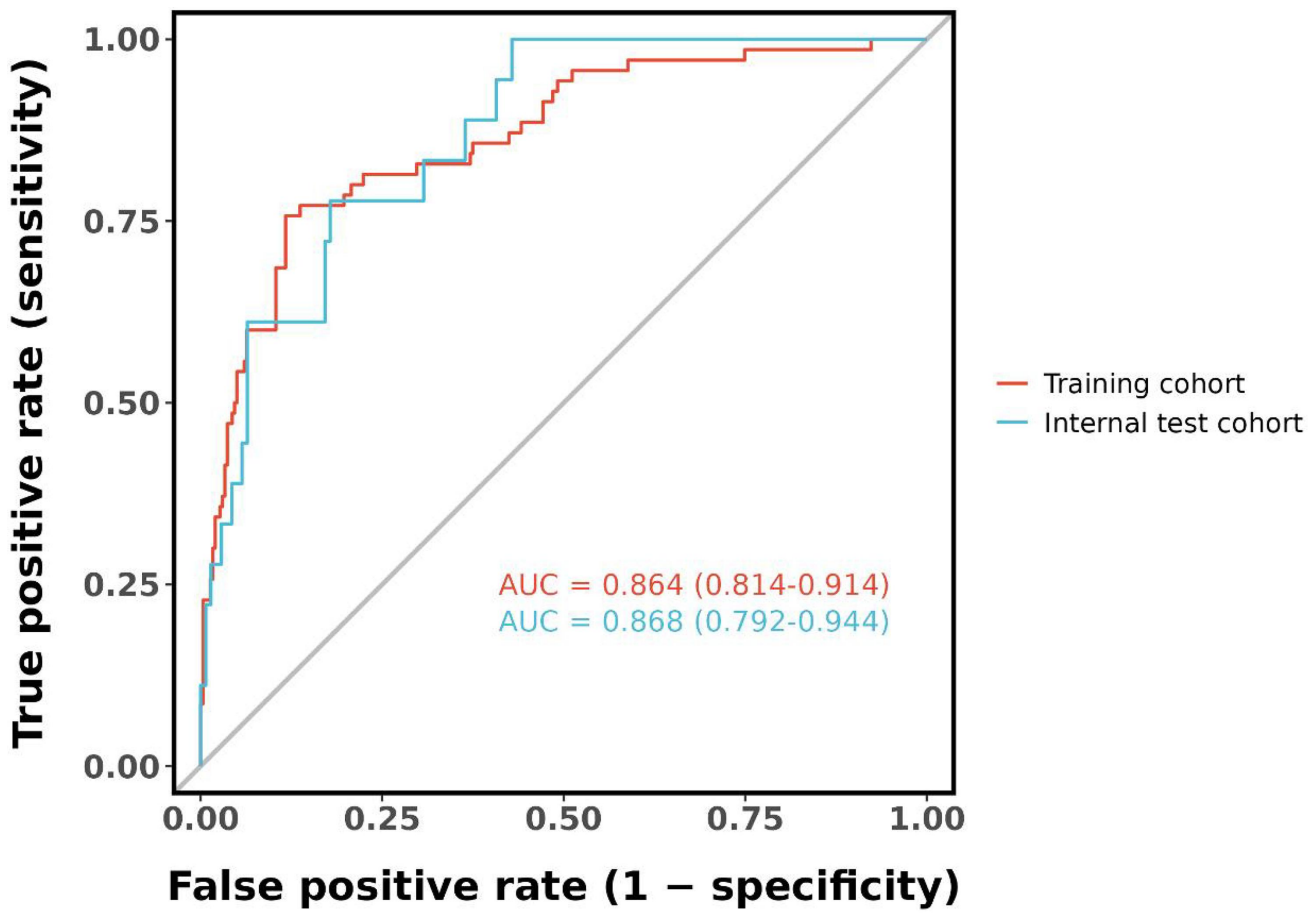
ROC curves of the nomogram in the training and internal validation cohorts.

The predictive performance of the nomogram was evaluated in both the training and internal validation cohorts. As shown in Figure 3, ROC analysis demonstrated good discrimination ability, with an area under the curve of 0.864 (95% CI, 0.814–0.914) in the training cohort and 0.868 (95% CI, 0.792–0.944) in the internal validation cohort, indicating that the nomogram had satisfactory predictive accuracy.

**Figure 4:**
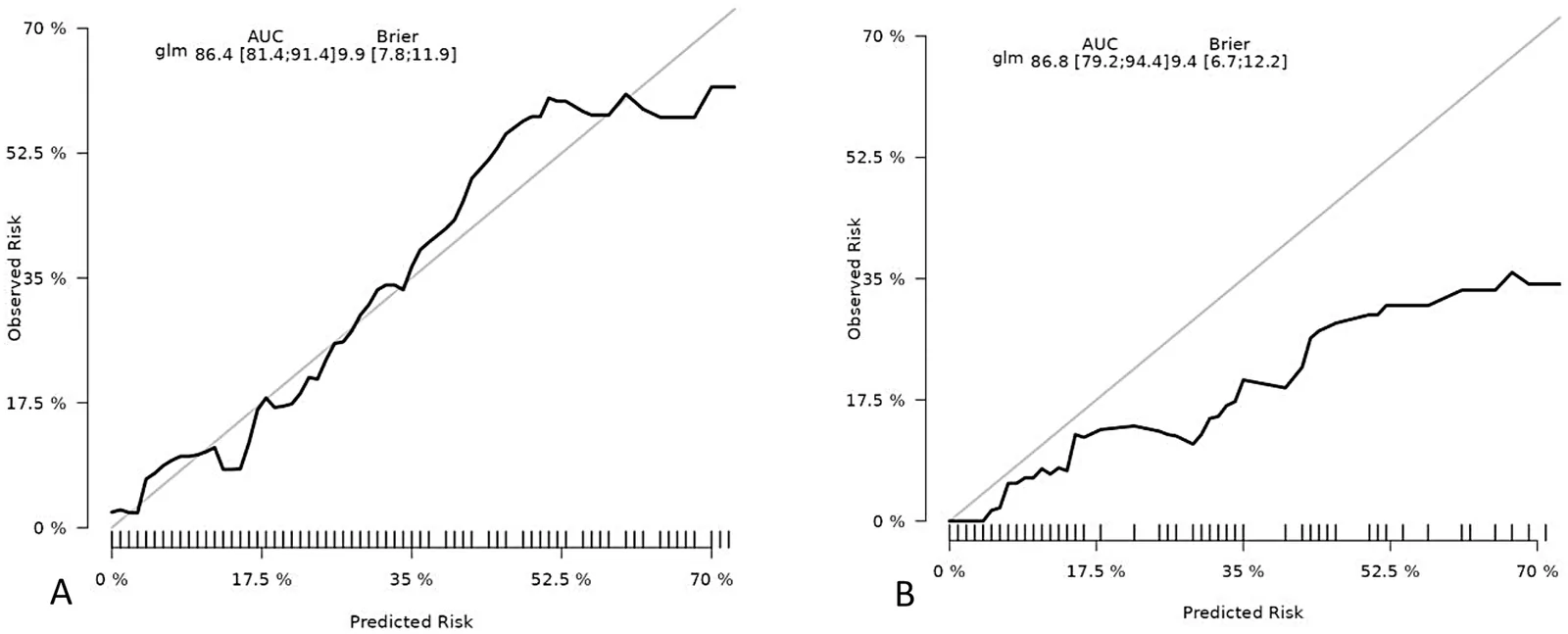
Calibration curves of the nomogram in the training and internal validation cohorts. [**(A)** For training cohort. **(B)** For internal validation cohort].

The calibration of the nomogram was further assessed using calibration curves. As shown in Figure 4, the predicted probabilities were in good agreement with the observed probabilities in both cohorts. The calibration curves were generated with 1,000 bootstrap resamples. The mean absolute error was 0.021 in the training cohort and 0.022 in the internal validation cohort, suggesting good calibration performance of the model.

**Figure 5:**
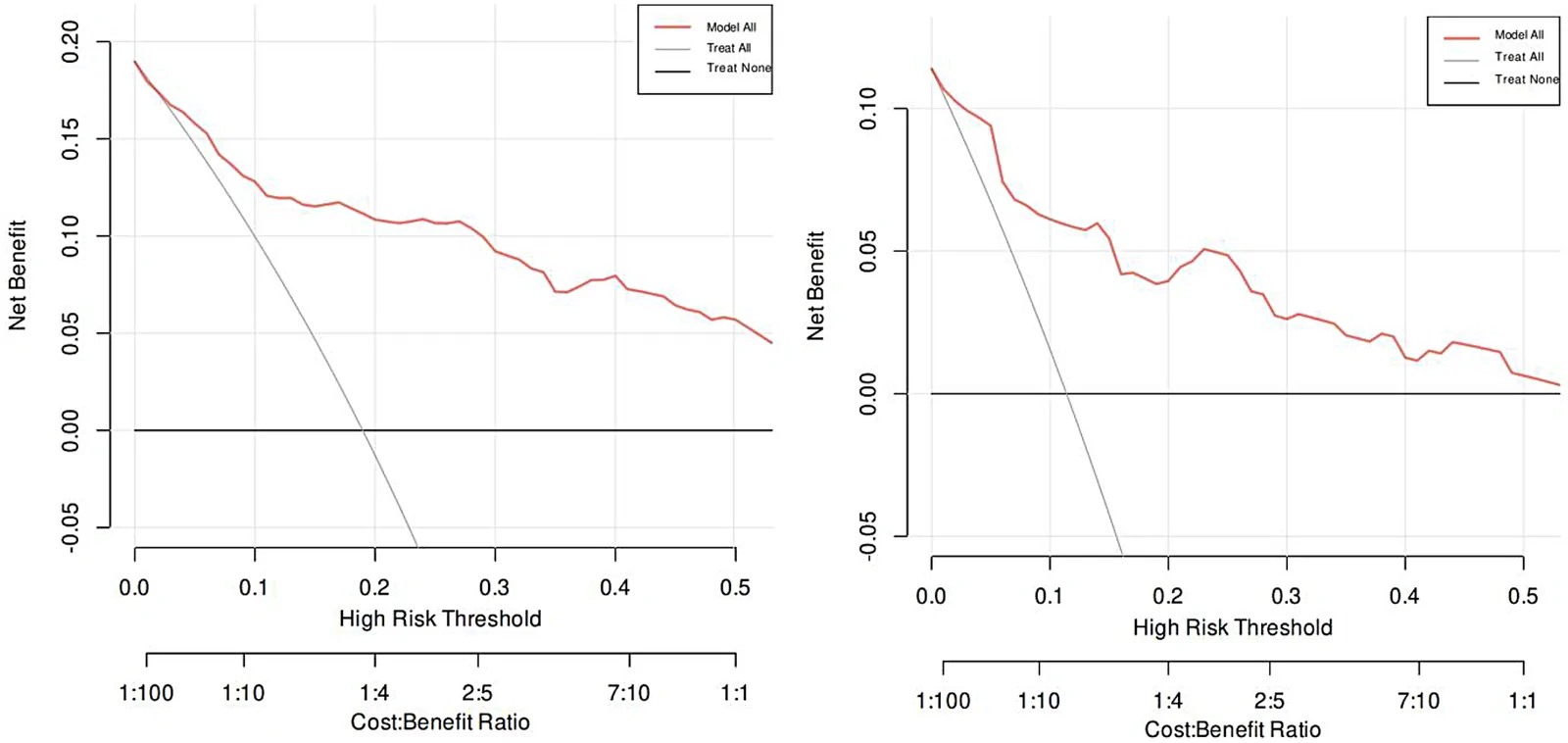
DCA of the nomogram in the training and internal validation cohorts. [**(A)** For training cohort. **(B)** For internal validation cohort].

The clinical utility of the nomogram was evaluated by DCA. As illustrated in Figure 5, the nomogram provided a positive net benefit across a wide range of threshold probabilities in both the training and internal validation cohorts, indicating its potential value in individualized clinical risk assessment and decision-making.

## Discussion

In the present study, we established and internally validated a nomogram for predicting postoperative hypotension following carotid endarterectomy. The model incorporated eight predictors selected from perioperative clinical and anatomical variables, namely hypertension, diabetes mellitus, plaque extension above the carotid bifurcation, symptomatic status, contralateral carotid stenosis, homocysteine, operation duration, and surgical treatment. The nomogram showed good discriminatory ability and calibration in both the training and internal validation cohorts, with favorable clinical net benefit demonstrated by decision curve analysis. Collectively, these results indicate that the nomogram may provide a convenient and individualized approach for perioperative risk stratification in patients undergoing carotid endarterectomy.

Carotid endarterectomy is one of the surgical intervention methods for treating carotid artery stenosis and is also an effective strategy for preventing stroke (10). Post-CEA hypotension is a postoperative hemodynamic complication of significant clinical importance, as it may damage cerebral and other vital organ perfusion in the early postoperative period. Its occurrence may be multifactorial, including dysfunction of blood pressure receptors due to stimulation of the carotid sinus surrounding area by surgical procedures, transient autonomic imbalance, anesthesia effects, changes in vascular reactivity, and surgical-related stress. Many patients require drug support (11). Clinically, especially in patients with limited cerebral vascular reserve or those with multiple comorbidities, postoperative hypotension is a serious burden (12). Therefore, establishing a reliable prediction model is valuable for identifying high-risk patients and optimizing perioperative management.

In our model, patients with a long-term history of hypertension before surgery had a lower risk of developing postoperative hypotension. This inverse association warrants further mechanistic consideration. Patients with chronic hypertension typically exhibit elevated baseline blood pressure, increased peripheral vascular resistance, reduced arterial compliance, and an upward resetting of the baroreflex set point (13). These characteristics may make it difficult for them to reach the diagnosis of hypotension in the early postoperative period. Furthermore, the chronic upregulation of sympathetic tone and the renin-angiotensin-aldosterone system in hypertensive patients may provide a physiological buffer against acute blood pressure drops, a phenomenon supported by previous studies on postoperative hemodynamic stability (14). The unopposed *α*-adrenergic vasoconstriction in chronically hypertensive patients may also contribute to greater resistance to vasodilatory stimuli during the early recovery phase. It is important to note, however, that the “protective” effect observed here is relative—patients with hypertension may experience absolute blood pressure reductions of similar magnitude, but because their baseline is higher, they are less likely to cross the hypotension threshold.

In contrast, diabetes remained a risk factor in the present model. This association may be related to cardiovascular autonomic nerve dysfunction, endothelial injury, and impaired vascular reactivity in patients with diabetes, all of which may increase the risk of hemodynamic instability during the perioperative period (15, 16). Patients with symptomatic carotid artery stenosis also have an increased risk of postoperative hypotension. This finding may suggest unstable plaques, which can cause transient cerebral ischemic attacks. These patients may have unstable hemodynamics and impaired compensatory capacity, making postoperative blood pressure more prone to fluctuation.

In this study, contralateral carotid artery stenosis was identified as an important predictor of postoperative hypotension. Although there is currently no direct evidence indicating a correlation between contralateral carotid artery stenosis and post-carotid endarterectomy hypotension, this factor may reflect more severe systemic atherosclerosis, impaired vascular compliance, and decreased hemodynamic adaptability, all of which may cause patients to experience unstable blood pressure after the surgery (17). Furthermore, bilateral carotid artery disease may indicate a more vulnerable overall circulatory state. Once hypotension occurs, it may be more likely to persist or require drug intervention. From a clinical perspective, due to the poorer cerebral collateral circulation for blood supply, these patients may be less tolerant of a decrease in blood pressure. Therefore, stenosis of the contralateral carotid artery may not only indicate a greater burden of atherosclerosis, but also suggest that the patient has a more vulnerable blood flow compensation capacity.

We also observed that elevated homocysteine levels were associated with a lower risk of postoperative hypotension, a finding that is unexpected and should be interpreted with caution. One plausible explanation is that hyperhomocysteinemia is associated with endothelial dysfunction, increased arterial stiffness, and reduced vascular compliance (18). These vascular alterations may result in elevated baseline peripheral vascular resistance and reduced sensitivity to acute vasodilatory stimuli, thereby offering relative protection against hypotension, similar to the pattern observed in chronic hypertension. However, this interpretation remains speculative. A more conservative interpretation is that this association may represent a chance finding due to the limited sample size or the presence of unmeasured confounders, such as preoperative medication use, nutritional status, or renal function. In addition, although homocysteine has been extensively studied as a risk factor for cardiovascular disease, its association with perioperative hemodynamic stability has not been well established in the literature. Therefore, this finding should not be overinterpreted or used to guide clinical management. Further studies with larger sample sizes are needed to validate this association and to elucidate the underlying mechanisms if it proves to be reproducible.

The method of surgical treatment was also an important component of the model. In particular, patch angioplasty showed a markedly higher odds ratio compared with eversion endarterectomy. This finding may suggest that different reconstruction techniques exert different effects on the carotid sinus region and postoperative blood pressure regulation. For example, greater traction, vessel wall dilation, or a wider reconstruction field may enhance baroreceptor-related hemodynamic responses (19, 20). However, previous studies have reported inconsistent findings regarding the relationship between surgical technique and postoperative blood pressure changes (21, 22). Therefore, this association should be interpreted cautiously and validated in further studies.

Plaque extension above the carotid bifurcation and operation duration were also incorporated into the nomogram, although their associations did not reach conventional statistical significance in the multivariable logistic regression model. These variables may still contribute incremental predictive information. In particular, plaque distribution above the bifurcation may influence the extent and location of surgical dissection, thereby affecting the degree of carotid sinus stimulation. Likewise, longer operation duration may reflect greater procedural complexity, more extensive manipulation, or increased perioperative physiological stress (23, 24). Therefore, from a predictive perspective, it is reasonable to include them in this nomogram. However, the underlying mechanism still needs to be further clarified.

Taken together, these findings highlight that baseline clinical characteristics, vascular anatomy, and procedural factors all play a role in the development of postoperative hypotension after carotid endarterectomy. The nomogram developed in this study showed good predictive performance in both the training and internal validation cohorts. The AUC values in the two cohorts were both greater than 0.86, indicating satisfactory discrimination, while the calibration curves showed good agreement between predicted and observed probabilities. In addition, decision curve analysis demonstrated a positive net benefit across a range of threshold probabilities, supporting the potential clinical applicability of the model. In practice, this nomogram may help clinicians identify patients at increased risk before surgery, enhance perioperative blood pressure surveillance, and guide individualized preventive or therapeutic strategies.

Several limitations of this study should be acknowledged. First, this was a retrospective single-center study, which may have introduced selection bias and limited the generalizability of the findings. Second, although we performed bootstrap resampling (1,000 repetitions) for calibration assessment and internal validation using a 7:3 random split, external validation in independent cohorts is still required to confirm the generalizability of our findings. Third, bootstrap-corrected discrimination estimates were not separately calculated. We acknowledge this as a limitation of the current validation approach, and future studies should consider bootstrap-corrected AUC estimates to further validate model performance. Finally, due to the observational design, detailed plaque calcification patterns were not routinely available on preoperative ultrasound in this retrospective cohort. Therefore, we were unable to include this variable in the present analysis. Future prospective studies incorporating detailed plaque morphology are needed to further validate the model.

Although some variables in the final model (plaque extension above the carotid bifurcation and operation duration) did not reach conventional statistical significance (*p* > 0.05) in the multivariable logistic regression, we retained them based on LASSO selection and their clinical relevance. LASSO regression is designed to perform simultaneous variable selection and regularization, and variables with non-zero coefficients may contribute incremental predictive information even if their individual *p*-values are not significant. Furthermore, plaque extension above the bifurcation reflects the extent of surgical dissection and carotid sinus manipulation, while operation duration serves as a surrogate for procedural complexity. The sensitivity analysis described above further supported the retention of these variables, as their exclusion led to a decrease in AUC, particularly in the validation cohort. Therefore, we retained all eight LASSO-selected variables in the final nomogram to balance statistical parsimony with clinical interpretability.

## Conclusion

We developed and internally validated a nomogram for predicting postoperative hypotension after carotid endarterectomy. The model demonstrated good discrimination, calibration, and potential clinical utility. Patients identified as high-risk through the nomogram may benefit from closer hemodynamic monitoring after surgery and earlier preparation for pharmacologic support.

## Data Availability

The original contributions presented in the study are included in the article/Supplementary Material, further inquiries can be directed to the corresponding author.
